# Detrimental effect of apoptosis of lymphocytes at an early time point of experimental abdominal sepsis

**DOI:** 10.1186/1471-2334-11-321

**Published:** 2011-11-20

**Authors:** Stefanos Atmatzidis, Ioannis M Koutelidakis, Grigorios Chatzimavroudis, Antigone Kotsaki, Konstantinos Louis, Aikaterini Pistiki, Athina Savva, Anastasia Antonopoulou, Konstantinos Atmatzidis, Evangelos J Giamarellos-Bourboulis

**Affiliations:** 1 2nd Department of Surgery, University of Thessaloniki, Medical School, Thessaloniki, Greece; 2 4th Department of Internal Medicine, University of Athens, Medical School, Athens, Greece

**Keywords:** apoptosis, abdominal sepsis, survival, lymphocytes, antimicrobials

## Abstract

**Background:**

Apoptosis of lymphocytes is considered a late sequelum in the sepsis cascade. The role of apoptosis of lymphocytes as a driver of final outcome was investigated.

**Methods:**

Abdominal sepsis was induced after cecal ligation and puncture (CLP) in 31 rabbits. Blood was sampled at serial time intervals and peripheral blood mononuclear cells (PBMCs) were isolated. Apoptosis of lymphocytes and monocytes was measured through flow cytometric analysis. PBMCs were stimulated with LPS and Pam3Cys for the release of tumor necrosis factor-alpha (TNFα). Tissue bacterial growth was quantitatively measured. In a second set of experiments, CLP was performed in another 40 rabbits; 20 received single intravenous infusions of ciprofloxacin and of metronidazole 4 hours after surgery.

**Results:**

Animals were divided into two groups based on the percentage of lymphocyte apoptosis at 4 hours after surgery; less than or equal to 32% and more than 32%. Survival of the former was shorter than the latter (p: 0.017). Tissue growth was similar between groups. Apoptosis of lymphocytes and of monocytes was lower in the former group over follow-up. Release of ΤNFα did not differ. The above findings on survival were repeated in the second set of experiments. Administration of antimicrobials prolonged survival of the former group (p: 0.039) but not of the latter group (pNS).

**Conclusions:**

Lymphocyte apoptosis at an early time point of experimental peritonitis is a major driver for death. A lower percentage of apoptosis leads earlier to death. Antimicrobials were beneficial even at that disease state.

## Background

Severe sepsis and septic shock are among the leading causes of death. It is estimated that almost 3 millions of cases appear annually in Northern America and in Europe; 35-50% of them die [1]. The abdomen is the second most common cause of sepsis in the European Intensive Care Units accounting for 26% of cases [2].

A general scheme of pathogenesis is well acceptable for all causes of sepsis. According to that scheme, sepsis is initiated when well-conserved microbial structures known as pathogen-associated molecular patterns (PAMPs) bind to receptors embedded either on the cell membranes or inside the cell cytoplasm of cells of the innate immune system, namely blood monocytes and tissue macrophages. These receptors are known as pattern recognition receptors (PRRs). Toll-like receptors (TLRs) are the best studied PRRs. Monomers of the peptidoglycan of the cell wall of Gram-positive cocci bind to TLR2 and lipopolysaccharides (LPS) of the outer membrane of Gram-negative bacteria bind to TLR4. The interaction of TLRs with PAMPs ends with the production of pro-inflammatory cytokines, like tumor necrosis factor-alpha (TNFα), interleukin (IL)-1β, IL-6 and IL-8. These pro-inflammatory mediators orchestrate septic reaction of the host. Soon after this first phase of hyper-production of pro-inflammatory mediators, a second phase ensues during which monocytes stimulated by PAMPs are no longer able to secrete a similar large amount of pro-inflammatory cytokines. Instead during this second phase a large amount of anti-inflammatory mediators like IL-10 are produced. This phase is considered a state of immunosuppression or immunoparalysis of the host when multiple organ dysfunctions take place [3]. Apoptosis of lymphocytes predominates during this stage and it is one of the main drivers leading to immunoparalysis [4].

However recent data of our group render questionable whether the above simplistic scheme may be generalized for severe sepsis/shock supervening in the field of all causes of sepsis. More precisely, flow cytometric analysis of monocytes and of lymphocytes was done within the first 24 hours upon diagnosis in 505 patients; 100 suffered from intra-abdominal infections [5]. Results revealed that advent of severe sepsis/shock in the event of abdominal sepsis differed considerably compared with sepsis originating from other sites. This was related with lower expression of HLA-DR on CD14-monocytes which is an index of immunoparalysis; greater counts of CD8-lymphocytes; and greater apoptosis of CD8-lymphocytes compared with other types of infections.

Based on the latter results it may also be hypothesized that all causes of abdominal sepsis are not similar in their ability to stimulate the host's immune response. Extensive work over the last two decades was done trying to simulate human abdominal sepsis with various animal models. The most widely applied models are that of intraperitoneal challenge with LPS or live bugs and that of cecal ligation and puncture (CLP) [6,7]. The present study aimed to investigate the importance of the apoptosis of lymphocytes early in the course of experimental peritonitis for the final outcome. The study used a model of intra-abdominal infection after CLP which mimics acute polymicrobial infections occurring in humans.

## Μethods

### Ethics Statement

The study received license permit K/8980/11-12-2006 form the Ethics Committee for Animal Experiments of the Perfecture of Athens. Permit was given in conformance with the following regulations: a) the Greek Presidential approval 30/1996; b) the ministerial decision 167/1997; c) the laws 1197/1981 and 2015/1992 about the rights and about protection of laboratory animals; and d) the directive 160/1991 of the European Union about performance of experiments in laboratory animals.

### Animals

A total of 75 white New Zealand male rabbits of a mean (± SD) weight of 3.19 ± 0.30 kg were studied. All animals were purchased from the same provider. They were transported in the animal house 10 days before operation for acclimatization. They were housed in single metal cages and had access to tap water and standard balanced rabbit chow *ad libitum*. Room temperature ranged between 18 and 22°C, relative humidity between 55 and 65% and the light/dark cycle was 6 am/6 pm.

### Model of peritonitis

The experiments were performed in two sets. In the first set, 35 animals were studied. The study endpoint of the initial set of experiments was the relationship between apoptosis of lymphocytes at 4 and 24 hours with 7-day survival. Animals were initially sedated by the intramuscular injection of 25 mg/kg of ketamine and 5 mg/kg of xylazine. Anesthesia was maintained by the intramuscular administration of 15 mg/kg of xylazine at 30-minute time intervals. In 31 rabbits, after an upper midline abdominal incision, the peritoneal cavity was entered and the intestines were displaced to the left. The cecum was recognized and ligated with a 3.0 suture. Three holes were performed in the wall of the cecum just above the suture with a 3.0 needle followed by light massage of the cecum. The peritoneal cavity and the abdominal wall were then closed in layers. Animal resuscitation was done by the continuous intravenous infusion of normal saline at a rate of 30 ml/hour through a catheter connected with the vein of the right ear. Four animals were sham-operated i.e. they were subject only to abdominal incision and closure. All experiments were performed on separate days by the same surgeons.

A volume of 5 ml of blood was sampled from the vein of the left ear of each animal under aseptic conditions before the operation, and at 4, 24 and 48 hours. Three ml were collected into heparin-coated tubes for flow cytometry and stimulation assays. One ml was collected into pyrogen-free tubes and centrifuged. Serum was kept refrigerated at -70°C for the measurement of tumor necrosis factor-alpha (TNFα). Another ml was added into tubes containing 4 ml of blood culture medium (Becton Dickinson, Cockeysville Md) and incubated at 35°C for seven days.

After the end of the operation, animals were transported to their cages. Survival was recorded every 12 hours for a total period of follow-up of 7 days. Every effort was done to minimize animal suffering. This was done by the administration of paracetamol suppositories twice daily starting two hours after the end of the operation. Within one hour after death autopsy was performed; under sterile conditions, segments from the right kidney, liver, spleen and lower lobe of the right lung were taken and placed into separate sterile plastic containers for quantitative cultures and biopsy. Animals remaining alive after 7 days were sacrificed. This was done after initial sedation followed by the rapid intravenous infusion of phenobarbital. Tissue cultures were drawn from these animals as described above.

In the second set of experiments, 40 rabbits were studied. In all animals, peritonitis was induced as described above; blood was sampled at 4 hours after induction of peritonitis. In 20 animals, single shots of ciprofloxacin and metronidazole were administered intravenously by one right ear vein at 4 hours after induction of peritonitis. The dose of ciprofloxacin was 30 mg/kg (Bayer, Germany) and that of metronidazole 25 mg/kg (Sanofi Aventis, France), as reported elsewhere [8,9]. Survival was recorded for 7 days.

The study endpoint was the relationship between apoptosis of lymphocytes at 4 and 24 hours with 7-day survival. To this end, Ethics Committees licensed the study since it was unavoidable to investigate the impact of apoptosis on survival without 7-day survival as an endpoint.

### Cell apoptosis and cell stimulation

**Figure 1:**
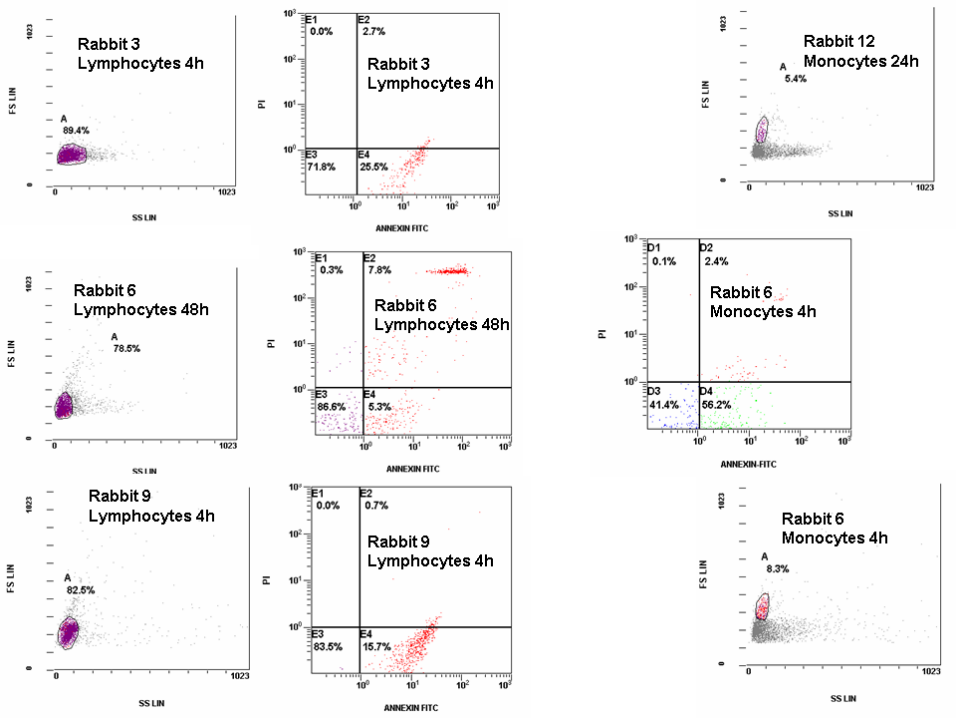
**Indicative forward and slide scattering of isolated mononuclear cells**. Separate gating for lymphocytes and monocytes was done according to their properties. Flow cytometry panels for apoptosis are also shown. The number of animals and the time of sampling are indicated.

PBMCs were isolated after gradient centrifugation of heparinized whole blood over Ficoll (Biochrom, Berlin, Germany). After three consecutive washings in ice-cold phosphate buffered saline pH 7.2 (Biochrom), PBMCs were counted in a Neubauer chamber after trypan blue exclusion of dead cells. Half of PBMCs were stained with the protein ANNEXIN-V at the flurochrome fluorescein isothiocyanate (FITC, emission 525 nm, Immunotech, Marseille, France) and with propidium iodine (PI) (emission 575 nm, Immunotech). Cells were analyzed after running through the EPICS XL/MSL flow cytometer (Beckman Coulter Co, Miami, Florida) with separate gating for lymphocytes and for monocytes based on their characteristic FS/SS scattering. Cells staining positive for ANNEXIN-V and staining negative for PI were considered apoptotic. Indicative gating on lymphocytes and on monocytes and staining for ANNEXIN-V and PI are shown in Figure 1.

The remaining half of PBMCs were distributed into wells of 96-well plate of a final volume of 0.2 ml per well with RMPI 1640 enriched with 10% Fetal Bovine Serum (Biochrom), 2 mM glutamine and 10 mM pyruvate at a density of 2 × 10^6 ^PBMCs/ml. They were stimulated without/with 10 ng/ml of LPS of *Escherichia coli *O155:H5 which is a TLR4 ligand (Sigma Co, St. Louis, USA) or without/with 5 μg/ml of Pam3Cys-SKKK (EMC Microcollections, Tübingen, Germany) which a TLR2 ligand. The plates were incubated for 24 hours at 37°C in 5% CO_2_. After incubation, plates were centrifuged and the supernatants were collected and stored at -70°C until assayed for TNFα. All stimulation assays were performed in duplicate.

### Bioassay for measurement of TNFα

TNFα was measured by a bioassay on L929 fibrosarcoma cell line, as already described [10,11]. Briefly, confluent cells were thoroughly washed with Hank' s solution and harvested with 0.25% thrypsin/0.02% EDTA (Biochrom). Cells were centrifuged, re-suspended in RMPI 1640 supplemented with 10% Fetal Bovine Serum and 2 mM of glutamine and distributed into a 96-well cell culture plate at a density of 1 × 10^5 ^cells/well. The final volume of fluid into each well was 0.05 ml. After incubation for 2 hours at 37°C at 5% CO_2_, 0.06 ml of supernatants or of serum or of standard dilutions of known concentrations of human TNFα (Sigma, range 5.75-375.00 pg/ml) were added into each well followed by 0.05 ml of a 0.3 mg/ml dilution of cycloheximide (Sigma) to inhibit de novo protein biosynthesis. After over-night incubation, the supernatant of each well was discarded by aspiration and 0.1 ml of a 0.5 mg/ml methylene blue solution in methanol 99% was added. After ten minutes, the dye was removed and wells were thoroughly washed three times with 0.9% sodium chloride. Wells were left to dry and remnants of the dye in each well became soluble by the addition of 0.1 ml of 50% glacial acetic acid (Merck, Darmstadt, Germany). Optical density in each well was read at 495 nm (Hitachi Spectophotometer, Tokyo, Japan) against blank wells and control wells without added serum. Concentrations of TNFα were estimated by the reduction of the optical density of control wells by unknown samples applying a standard curve generated by standard concentrations. All determinations were performed in quadruplicate. The inter-day variation of the assay was 13.75%.

### Tissue cultures

Tissue segments were weighted and homogenized; one 0.1 ml aliquot was diluted 1:10 into sterile sodium chloride four consecutive times. Another aliquot of 0.1 ml of each dilution was plated onto MacConkey agar and incubated at 35°C for a total period of three days. Plates were incubated at 35°C and the number of viable colonies were counted into each dilution and multiplied by the appropriate dilution factor. Identification of bacteria was performed by the API20E and the API20NE systems (bioMerieux, Paris, France). The lower detection limit was 30 cfu/g. Bacterial cells were expressed by their log_10 _value.

### Statistical analysis

Results were expressed by their mean (± SE). Comparisons between groups were performed by Mann-Whitney U test. Survival was estimated by Kaplan-Meier analysis; groups were compared by the log-rank test. Bacterial growth was assessed separately for animals that died and separately for animals that were sacrificed after 7 days. To define if the time until death may have an impact on the results of tissue cultures, correlation according to Spearman's rank of order was done between time until death and tissue bacterial growth. Any value of p below 0.05 was considered significant after adjustment for multiple comparisons.

## Results

Mean ± SE apoptosis of lymphocytes of the four sham-operated rabbits and of the 31 rabbits with induced peritonitis were 2.4 ± 0.9% and 25.3 ± 3.8% respectively at 4 hours (p: 0.007); 1.1 ± 0.6% and 19.5 ± 4.6% at 24 hours (p: 0.037); and 2.4 ± 0.7% and 21.2 ± 4.3% at 48 hours (p: 0.032). Respective apoptosis of monocytes were 36.9 ± 10.8% and 42.7 ± 4.3% at 4 hours (p-non significant); 13.7 ± 2.9% and 41.6 ± 5.4% at 24 hours (p: 0.042); and 27.8 ± 11.8% and 45.9 ± 6.1% at 48 hours (p: 0.039).

**Figure 2:**
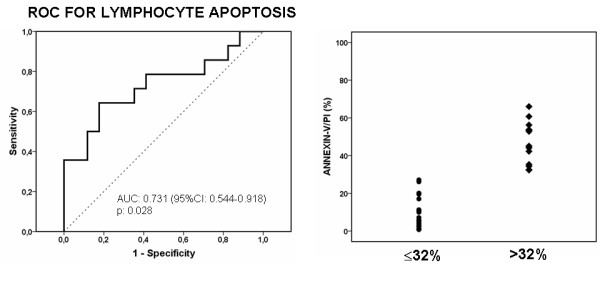
**Receiver operator curve (ROC) analysis of apoptosis of lymphocytes at 4 hours to predict survival among rabbits subject to experimental peritonitis (left panel)**. According to results of ROC analysis rabbits were divided into two groups; those with apoptosis ≤32% and those with apoptosis > 32%. The scatterplot of lympchocyte apoptosis is shown in the right panel.

ROC analysis was performed within rabbits subject to the first set of experimental peritonitis for apoptosis of lymphocytes and of monocytes at 4 hours and at 24 hours as predictors of death. The only analysis yielding statistical significance was apoptosis of lymphocytes at 4 hours (AUC: 0.731, 95%CI: 0.544-0.918, p: 0.028). Apoptosis of lymphocytes lower than or equal to 32% had specificity 80% to predict death (Figure 2). This specificity cut-off has been considered of significance for biomarkers to define an event in sepsis studies [12].

**Figure 3:**
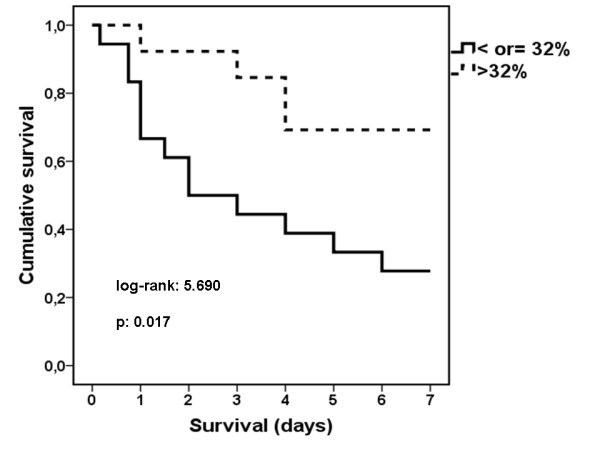
**Comparative survival of rabbits subject to experimental peritonitis**. Animals are divided into two groups based on the percentage of apoptosis of lymphocytes 4 hours after induction of peritonitis: ≤32% or > 32%.

Animals were divided into two groups according to that finding: i) those with lymphocyte apoptosis less than or equal to 32%. This was found in 18 animals; and ii) those with lymphocyte apoptosis greater than 32% (Figure 2). This was found in 13 animals. Thirteen (72.2%) and four (20.8%) of them died respectively (p: 0.017, Figure 3).

We hypothesized that at least two explanations may exist behind that phenomenon: a) apoptosis of lymphocytes of the first 4 hours may influence bacterial replication in tissues; and/or b) apoptosis of lymphocytes may determine death by an immune-related mechanism.

**Figure 4:**
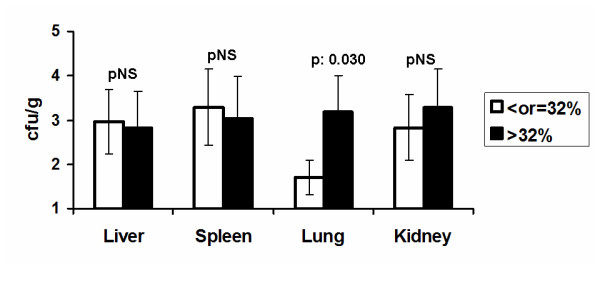
**Tissue bacterial growth in liver, spleen, right kidney and lower right lung lobe of rabbits subject to experimental peritonitis**. Animals are divided into two groups based on the percentage of apoptosis of lymphocytes 4 hours after induction of peritonitis: ≤32% or > 32%. NS: non-significant.

Blood cultures were sterile. Bacterial growth was measured in the liver, spleen, lung and kidneys of animals after death or sacrifice. No significant correlation was found between time to death and tissue bacterial growth in any tested organ. Bacterial species isolated from tissues of animals were *Escherichia coli*, *Enterobacter cloacae *and enterococci. No differences of tissue bacterial growth were found between animals with apoptosis of lymphocytes at 4 hours lower than or equal to 32% and animals with apoptosis of lymphocytes at 4 hours greater than 32% in the liver, in the spleen and in the kidney. However, bacterial growth in the lung was greater within animals with apoptosis of lymphocytes at 4 hours greater than 32% (Figure 4).

**Figure 5:**
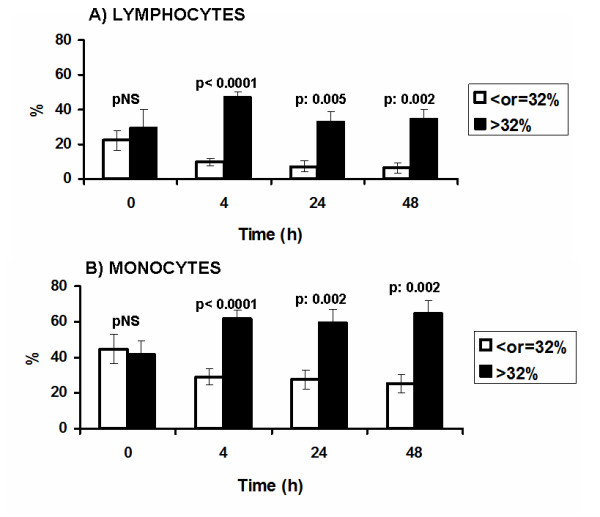
**Comparative apoptosis of lymphocytes and of monocytes over consecutive follow-up of rabbits subject to experimental peritonitis**. P values refer to comparisons between animals with percentages of apoptosis of lymphocytes 4 hours after induction of peritonitis either ≤32% or > 32%; NS: non-significant.

The impact of lymphocyte apoptosis on the immune response of the host involved a study on a) monocytes apoptosis; and b) the innate immune response. Apoptosis of lymphocytes and of monocytes over follow-up is shown in Figure 5. At that analysis animals are divided into two groups; those with lymphocyte apoptosis ≤32% at 4 hours and those with lymphocyte apoptosis > 32% at 4 hours. At baseline i.e. before induction of peritonitis, the two animal groups did not differ. However, during the entire follow-up after induction of peritonitis, apoptosis of lymphocytes and apoptosis of monocytes were significantly greater in the group of animals with lymphocyte apoptosis > 32% at 4 hours (Figure 5).

**Figure 6:**
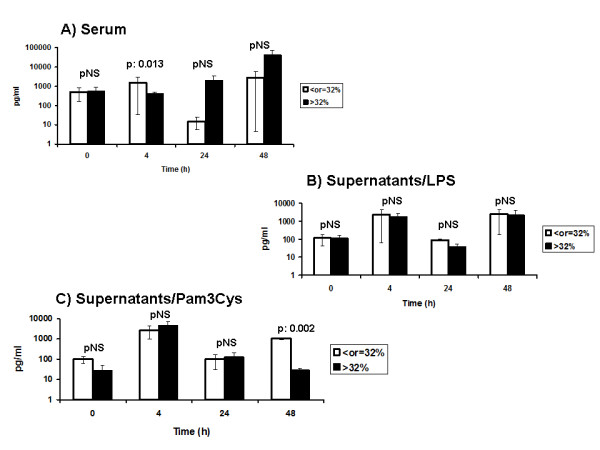
**Comparative concentrations of tumor necrosis factor-alpha (TNFα) in serum and in supernatants of peripheral blood mononuclear cells isolated over consecutive follow-up of rabbits subject to experimental peritonitis after stimulation with LPS or Pam3Cys**. P values refer to comparisons between animals with percentage of apoptosis of lymphocytes 4 hours after induction of peritonitis either ≤32% or > 32%; NS: non-significant.

TNFα produced by PBMCs of these two groups after stimulation with LPS and Pam3Cys generally did not differ with the exception of 48 hours. At that time point, TNFα stimulated by Pam3Cys was greater within the group of animals with lymphocyte apoptosis ≤32% at 4 hours. Serum TNFα differed at 4 hours after induction of peritonitis; it was greater among the group of animals with lymphocyte apoptosis ≤32% at 4 hours (Figure 6).

One question needing clarification was the cause of animal death. Although post-mortem histology was not done, tissue bacterial growth of intraperitoneal organs i.e. liver and spleen was much greater among non-surviving animals at 7 days than among surviving at 7 days. More precisely, mean ± SE log_10 _of bacteria in the liver of non-surviving animals at 7 days were 3.81 ± 0.75 cfu/g; among surviving animals at 7 days they were 1.98 ± 0.67 cfu/g (p: 0.045). Respective counts of bacteria in the spleen were 4.48 ± 0.96 cfu/g and 1.86 ± 0.61 (p: 0.031). However, tissue bacterial growth of extraperitoneal organs i.e. lung and kidney did not differ between non-surviving and surviving animals at 7 days. More precisely, mean ± SE log_10 _of bacteria in the lung of non-surviving animals at 7 days were 2.78 ± 0.71 cfu/g; among surviving animals at 7 days they were 1.96 ± 0.56 cfu/g (p non-significant). Respective counts of bacteria in the kidney were 3.92 ± 0.88 cfu/g and 2.16 ± 0.63 (p non-significant). These findings allowed identify abdominal sepsis as the cause of animal death.

**Figure 7:**
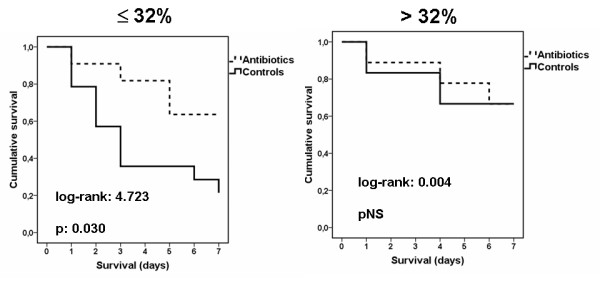
**Effect of administration of single doses of ciprofloxacin and of metronidazole 4 hours after induction of experimental peritonitis in survival**. Animals are divided into two groups based on the percentage of apoptosis of lymphocytes 4 hours after induction of peritonitis: ≤32% or > 32%.

From the 20 untreated rabbits studied in the second set of experiments, apoptosis of lymphocytes at 4 hours was ≤32% in 14 rabbits and > 32% in six rabbits; 11 (78.6%) and two of them died (33.3%) respectively. This finding replicated results of the first set of experiments. From the 20 animals administered single shots of ciprofloxacin and metonidazole at 4 hours after induction of peritonitis, 11 animals had lymphocyte apoptosis at 4 hours before antibiotic infusion ≤32% and nine animals had lymphocyte apoptosis at 4 hours before antibiotic infusion > 32%. Four (27.4%) and three (23.3%) of them died respectively. Comparison of survival with the respective non-antibiotic treated animals is shown in Figure 7. It was evident that infusion of antibiotic prolonged survival of animals with lymphocyte apoptosis at 4 hours ≤32% but not of animals with lymphocyte apoptosis at 4 hours > 32%.

## Discussion

Sepsis is a syndrome characterized by great heterogeneity. Although all patients are ubiquitously characterized as septic, they greatly differ regarding gender, age, co-morbid conditions, underlying infections, and offending pathogens. On that sense, it may be hypothesized that intra-abdominal infections differ from other types of infections in the way they trigger sepsis cascade [13]. Some of them develop in the event of another intrabdominal sterile critical illness like acute pancreatitis. Some develop due to slow seeding of bacterial gut content in the circulation like an abscess or acute diverticulitis. Others develop due to the acute and abrupt leakage of large amounts of the bowel content like in acute peritonitis after gut rupture [6,14,15]. Although all these cases are polymicrobial in nature, they may differ in the type of stimulation of the immune system of the host.

A model of CLP is used in the present study attempting to evaluate the effect of bacteria load released from the gut in the event of intra-abdominal sepsis on the innate and adaptive immune responses of the host. The study of rabbits instead of rodents allows better reproducibility of the experimental conditions. Reproducibility is one major factor creating diversity in such type of experiments [7]. Rabbits are larger animals than mice. This limits a) diversities created by the surgical team within separate days of experiments; and b) the impact of sequential blood sampling on survival. Although recent evidence suggest that daily sampling of volumes as high as 35 μl in mice subject to CLP do not affect mortality [16], the usage of rabbits remains indispensable for the present study where daily sampling of volumes as high as 5 ml is necessary to collect the necessary amount of PBMCs.

Apoptosis of lymphocytes is conceived as sequelea of severe sepsis [17,18]. In accordance with that an antibody against receptor PD-1 (programmed-death-1), which is a negative co-stimulatory molecule on T-lymphocytes, was administered in mice late after induction of CLP and sepsis. Treatment prevented apoptosis of T-lymphocytes and prolonged survival [19]. However, recent evidence shows that apoptosis of T-lymphocytes may take place early over the course of sepsis [20] and a new important role has been suggested for apoptosis of T lymphocytes to regulate the innate immune response [21].

Our findings corroborate with previous results showing that apoptosis of lymphocytes is taking place in sepsis since apoptosis of lymphocytes was greater in rabbits subject to CLP and sepsis than sham-operated rabbits at all times of sampling. However, the presented results indicate that the rate of lymphocyte apoptosis as early as 4 hours after induction of peritonitis may define final outcome. The importance of this novel concept is underscored by the efficacy of treatment outcome. More precisely, administration of antimicrobials improved survival of animals that preserved their lymphocytes at 4 hours after induction of peritonitis. The efficacy of antimicrobials was limited in those animals with a great level of apoptosis. These findings may suggest that factors other than proper care impact on outcome from sepsis. However, it is obvious that the current findings support like never before the need for a very early start of antimicrobials and for early debridement of an intra-abdominal infection, as already suggested in the current treatment guidelines [22].

The mechanism of regulation of final outcome by apoptosis of lymphocytes taking place early over the time course of sepsis is difficult to interpret. According to the present findings, apoptosis of monocytes is also affected and this may influence phagocytosis. Other authors suggest that the rate of apoptosis of T lymphocytes in sepsis may have a direct impact on the process of phagocytosis [21].

Two main limitations of the present study should be addressed: a) the lack of commercially-available specific antibodies allowing discrimination of blood lymphocytes from blood monocytes in the rabbit. However, their physical properties of forward scattering and side scattering allow safe discrimination of these two populations; and b) the lack of measurement of the precise lymphocyte count at every time of sampling. Although this may be of importance, it could hardly affect the presented results that are focusing on the apoptotic process per se.

The precise trigger of lymphocyte apoptosis early over the course of sepsis is unknown. In a recent publication of our group, one probable explanation is described. More precisely, serum was isolated from 48 patients with severe sepsis/shock with the first 12 hours from advent of organ failures. When CD4-lymphocytes isolated from healthy volunteers were incubated with patients' serum, apoptosis was induced after activation of both the extrinsic and the intrinsic apoptotic pathways. This was not the case whenever CD4-lymphocytes were incubated with healthy serum. These findings may be interpreted in two ways: either an inhibitor of apoptosis circulates normally which is down-regulated early in severe sepsis; or a circulatory factor stimulating apoptosis appears early in severe sepsis [23].

## Conclusions

The presented results revealed that lymphocyte apoptosis at an early time point of experimental peritonitis was a major driver for death. A lower percentage of lymphocyte apoptosis within the first 4 hours from the induction of the septic process led earlier to death. Antimicrobials could be beneficial even at that state underscoring the significance of early administration of antimicrobial therapy for the septic patient with intra-abdominal infection. These findings create the need for similar studies in other models of sepsis.

## List of abbreviations

CLP: cecal ligation and puncture; FITC: fluorescein isothiocyanate; LPS: lipopolysaccharide; IL: interleukin; PAMP: pathogen-associated molecular patterns; PBMCs: peripheral blood mononuclear cells; PI: propidium iodine; PRR: pattern recognition receptor; TLR: Toll-like receptor; TNFα: tumour necrosis factor-alpha

## Competing interests

The authors declare that they have no competing interests.

## Authors' contributions

SA performed animal experiments, participated in data analysis and read and approved the final manuscript. IK and GC participated in animal experiments and read and approved the final manuscript. AK and AP performed flow cytometry analysis, isolation and culture of PBMCs and read and approved the final manuscript. KL, AS and AA performed tissue cultures and cytokine measurements and read and approved the final manuscript. KA designed the study and read and approved the final manuscript. EJGB performed analysis and wrote and approved the final manuscript

## Pre-publication history

The pre-publication history for this paper can be accessed here:

http://www.biomedcentral.com/1471-2334/11/321/prepub
